# Modeling lactate threshold in cycling—influence of sex, maximal oxygen uptake, and cost of cycling in young athletes

**DOI:** 10.1007/s00421-025-05744-y

**Published:** 2025-04-12

**Authors:** Jonas Fischer, Finn Hävecker, Sanghyeon Ji, Patrick Wahl, Sebastian Keller

**Affiliations:** 1 Department of Exercise Physiology, German Sport University, Cologne, Germany; 2 German Research Centre of Elite Sport, German Sport University, Cologne, Germany

**Keywords:** Maximal metabolic steady state, Performance diagnostics, Aerobic capacity, Endurance performance, Gross efficiency, Maximal lactate accumulation rate, Youth athletes

## Abstract

**Purpose:**

Understanding physiological determinants of lactate threshold 2 (LT2) is crucial for tracking adaptations and deriving individualized training recommendations in cycling. Therefore, the study investigated: 1. the accuracy of modeling power output at LT2 in young athletes of both sexes using maximal oxygen uptake (${\dot{V}}\textrm{O}_{2_\textrm{peak}}$), fractional utilization of ${\dot{V}}\textrm{O}_{2_\textrm{peak}}$ (%${\dot{V}}\textrm{O}_{2_\textrm{peak}}$), and oxygen cost of cycling (Cc); 2. the influence of Cc determination on the model accuracy; 3. the influence of the model predictors and inclusion of maximal lactate accumulation rate (${\dot{c}}La_\textrm{max}$) on power at LT2 depending on sex.

**Methods:**

Eighty-three cyclists and triathletes (22 females, 61 males; age [median and IQR]: 14.6 [13.8–17.6] years, ${\dot{V}}\textrm{O}_{2_\textrm{peak}}$ [mean ± SD]: 59.2 ± 6.5 mL⋅kg^–1^⋅min^–1^) performed an incremental test to determine power at LT2, ${\dot{V}}\textrm{O}_{2_\textrm{peak}},$ %${\dot{V}}\textrm{O}_{2_\textrm{peak}}$ at LT2, and Cc (assessed at 3 W⋅kg^–1^, 75% ${\dot{V}}\textrm{O}_{2_\textrm{peak}},$ and 90% LT2).

**Results:**

Modeled and experimentally determined power at LT2 demonstrated excellent agreement for all, male and female athletes (ICC $\ge$ 0.961), with Cc at 90% LT2 providing the highest accuracy (ICC $\ge$ 0.986). The three physiological determinants explained $\ge$ 98% of the variance in power at LT2, with the largest unique contribution from ${\dot{V}}\textrm{O}_{2_\textrm{peak}}$ (62 and 67% of total $R^2$), followed by Cc (8 and 34%) and %${\dot{V}}\textrm{O}_{2_\textrm{peak}}$ at LT2 (5 and 12%) in males and females, respectively, while ${\dot{c}}La_\textrm{max}$ did not improve the regression.

**Conclusion:**

${\dot{V}}\textrm{O}_{2_\textrm{peak}},$ %${\dot{V}}\textrm{O}_{2_\textrm{peak}}$ at LT2 and Cc accurately predict power at LT2 in young cycling athletes independent of sex, with determination of Cc at 90% LT2 providing the highest accuracy. While ${\dot{V}}\textrm{O}_{2_\textrm{peak}}$ contributes most to LT2 in both sexes, Cc appears more important in young females.

## Introduction

Quantifiable parameters showing the performance capabilities of athletes are crucial for the analysis and diagnostics of endurance sports. Important physiological parameters were shown to be maximal oxygen consumption (${\dot{V}}\textrm{O}_{2_\textrm{peak}}$), fractional utilization of ${\dot{V}}\textrm{O}_{2_\textrm{peak}}$ (%${\dot{V}}\textrm{O}_{2_\textrm{peak}}$), and exercise economy (C) (Joyner and Coyle 2008; McLaughlin et al. 2010). ${\dot{V}}\textrm{O}_{2_\textrm{peak}}$ represents the maximal rate of oxidative energy supply and, together with the glycolytic and phosphagen system, determines maximal metabolic power (Capelli 1999). In contrast, %${\dot{V}}\textrm{O}_{2_\textrm{peak}}$ displays the proportion of oxygen uptake (${\dot{V}}\textrm{O}_{2}$) required for a submaximal exercise intensity, e.g., at lactate threshold 2 (LT2), relative to ${\dot{V}}\textrm{O}_{2_\textrm{peak}}$ (Joyner and Coyle 2008), while C describes the ${\dot{V}}\textrm{O}_{2}$ required to generate a certain external work rate (Lundby et al. 2017). In cycling, the latter is often expressed as oxygen cost of cycling (Cc [mL⋅min^–1^⋅W^–1^]), calculated by dividing ${\dot{V}}\textrm{O}_{2}$ by the respective mechanical power output (Støren et al. 2014). Alternatively, it is expressed as gross efficiency (GE [%]), calculated by dividing mechanical power output by metabolic power input (Hopker et al. 2010). Consequently, Cc and GE have an inverse mathematical relationship (i.e., a higher GE indicates lower Cc). The relevance of the cited parameters for endurance performance has been demonstrated in the physiological model first introduced by Costill et al. (1971) and later popularized by Joyner (1991) (Eq. 1). For example, using this model, a high predictive validity has been shown for 16-km time trial performance in well-trained runners (*R*^2^ = 0.95) (McLaughlin et al. 2010) and for 15-km time trial performance in trained cyclists (*R*^2^ = 0.85) (Støren et al. 2013).

1 $$\begin{aligned} \text {Endurance performance} \, = \, \%{\dot{V}}\textrm{O}_\textrm{2peak} \cdot \frac{{\dot{V}}\textrm{O}_\textrm{2peak}}{C} \end{aligned}$$

One of the best single estimates of endurance performance is work rate (e.g., speed or power output) corresponding to the maximal metabolic steady state as a bioenergetic threshold, which is characterized by the highest equilibrium in blood lactate levels (bLa), i.e., maximal lactate steady state (MLSS), and ${\dot{V}}\textrm{O}_{2}$ and presents the upper boundary of the heavy intensity domain (Iannetta et al. 2021; Faude et al. 2009; Caen et al. 2024). Determining maximal metabolic steady state requires multiple 30-min constant work rate tests over several days, making it time consuming. As an alternative, various estimation methods use bLa kinetics from incremental tests to identify the bioenergetic threshold LT2 (Faude et al. 2009; Caen et al. 2024). However, these have led to heterogeneous results in terms of concurrent validity (i.e., agreement with MLSS as a bioenergetic criterion) and predictive validity (i.e., correlation with performance). In addition, besides representing MLSS and endurance performance as precisely as possible, power output or bLa at LT2 alone does not provide any information about the underlying physiological characteristics, i.e., ${\dot{V}}\textrm{O}_{2_\textrm{peak}}$ or Cc, which, however, can be relevant to derive individual training recommendations (Niemeyer et al. 2022).

Such differences in individual physiology can be due, among others, to different ages (i.e., when comparing junior and adult athletes) or sex. For example, a greater proportion of type I fibers and a greater oxidation rate of pyruvate were observed in young athletes compared to adults (Boisseau and Delamarche 2000). Due to the potential influence on the rate of glycolysis and thus on bLa kinetics on which the model parameter %${\dot{V}}\textrm{O}_{2_\textrm{peak}}$ is based, the accuracy of the entire model could also be affected. With respect to sex, it has recently been shown that differences in model parameters such as ${\dot{V}}\textrm{O}_{2_\textrm{peak}}$ and C can also differently affect the outcome of the model, in this case running speed at LT2 (Støa et al. 2020; Ji et al. 2023). While high variance explanation was reported for both adult male and female runners of different performance levels (${R}^{2}$ $\ge$ 0.88) by Støa et al. (2020), male runners were characterized by a higher ${\dot{V}}\textrm{O}_{2_\textrm{peak}}$ and females by a better (i.e., lower) C, which also correlated more strongly with running speed at LT2 in females than in males. In line with these results, Ji et al. (2023) also found higher ${\dot{V}}\textrm{O}_{2_\textrm{peak}}$ in young male compared to female squad athletes, whereas the latter showed better C, which was also more important for running speed at LT2. While the accuracy of the model was already demonstrated by a very high correlation with power output at LT2 in well-trained adult male cyclists (${R}^{2}$ = 0.95) (Støren et al. 2014), its applicability to young and female athletes still needs to be investigated to use it for individual training derivations.

Concerning the methodological determination of the model parameters, it has been suggested that Cc and GE in cycling are not fixed, but show inter-individual differences (Scharhag-Rosenberger et al. 2010; Sidossis et al. 1992). Further, it has been demonstrated that the assessment of C relative to a bioenergetic threshold resulted in comparable metabolic demands between participants regardless of the type of exercise (Sabater-Pastor et al. 2022) and that this approach likewise allowed a more accurate estimation of running speed at LT2 (Ji et al. 2023). Therefore, to determine whether such inter-individual differences and the assessment of Cc at different work rates influence the model, different methods will be compared in the present study to evaluate the agreement of modeled and experimentally determined power output at LT2, consistent with previous work (Ji et al. 2023). Besides the model predictors, the criterion used to determine the model estimate (i.e., power output at LT2) likely also influences the predictive validity (Ji et al. 2023). Since no validated LT2 determination method has been used in cycling so far, this will be done for the first time in the present study (Zwingmann et al. 2019). In addition, due to the current research interest in glycolytic rate, especially in cycling (e.g., Haase et al. 2024; Yang et al. 2024; Quittmann et al. 2020), the influence of maximal lactate accumulation rate (${\dot{c}}La_\textrm{max}$) on power output at LT2 is also tested. Despite a rather small contribution to the variance explained in 5-km running performance (4.4%) (Quittmann et al. 2023), ${\dot{c}}La_\textrm{max}$ is theoretically attributed a direct influence on power output at LT2 (i.e., the higher the ${\dot{c}}La_\textrm{max}$, the lower the power at LT2, as long as all other determinants are constant) (Wackerhage et al. 2022).

In summary, we aimed to investigate in the present study: 1. the accuracy of the model compared to a validated determination of LT2 in cycling in young trained athletes depending on sex; 2. the influence of Cc determination method on the accuracy of the model; 3. the influence of the model predictors and the addition of ${\dot{c}}La_\textrm{max}$ on power output at LT2 depending on sex.

## Materials and methods

### Participants

The study sample consisted of young cyclists or triathletes from the federal state of North Rhine-Westphalia (Germany) who participated in a routine performance examination (*N* = 154) and were classified as trained according to De Pauw et al. (2013). All participating athletes and their parents or legal guardians provided written informed consent. The experimental testing was approved by the local ethics committee (approval number: 67/2020) and complies with the Declaration of Helsinki. In summary, 71 tests were excluded because at least one of the following criteria ensuring validity and comparability was not met: (a) age < 22 years (one test); (b) volitional exhaustion at the end of the incremental test (six tests); (c) valid determination of LT1 and LT2 using the modified maximal deviation method (see below) (five tests). In addition, only one valid data set was included in case of multiple visits (33 tests) and erroneous or incomplete data were removed (26 tests). Finally, 83 athletes (22 females and 61 males; 36 triathletes and 47 cyclists) who met all the criteria were included in this study. An overview of anthropometric and physiological characteristics is provided in Table 1.

**Table 1:** Descriptive anthropometric and physiological characteristics (mean ± standard deviation for normally distributed and median and interquartile range for non-normally distributed variables) of participants

Variable	All (*N* = 83)	Males (*N* = 61)	Females (*N* = 22)	*p* (m vs. f)
Anthropometrics				
Age [y]	14.6 (13.8-17.6)	14.3 (13.8-17.4)	15.8 (13.8-17.6)	> 0.05$^{\textrm{w}}$
Height [cm]	171 ± 9	173 ± 9	166 ± 5	< 0.001
Body mass [kg]	59.0 ± 9.4	60.1 ± 10.2	56.2 ± 6.1	< 0.05
Peak power output				
[W]	272 ± 56	286 ± 58	235 ± 26	< 0.001
[W⋅kg^–1^]	4.6 ± 0.5	4.8 ± 0.5	4.2 ± 0.4	< 0.001
Maximal oxygen uptake				
[mL⋅min^–1^]	3496 ± 677	3677 ± 677	2991 ± 342	< 0.001
[mL⋅kg^–1^⋅min^–1^]	59.2 ± 6.5	61.3 ± 5.6	53.4 ± 5.1	< 0.001
Oxygen cost of cycling				
${\textrm{Cc}}_{\textrm{fix}}$ [mL⋅min^–1^⋅W^–1^]	13.6 ± 0.9	13.7 ± 0.8	13.2 ± 0.9	< 0.05
${\textrm{Cc}}_{\%{\dot{V}}\textrm{O}_{2_\textrm{peak}}}$ [mL⋅min^–1^⋅W^–1^]	13.3 ± 1.0	13.4 ± 1.0	13.2 ± 1.1	> 0.05
${\textrm{Cc}}_{\%{\rm LT}2}$ [mL⋅min^–1^⋅W^–1^]	13.3 ± 0.9	13.3 ± 0.9	13.2 ± 1.0	> 0.05
Gross efficiency				
GE_fix_ [%]	21.1 ± 1.3	20.9 ± 1.2	21.6 ± 1.5	< 0.05
${\textrm{GE}}_{\%{\dot{V}}\textrm{O}_{2_\textrm{peak}}}$ [%]	21.5 ± 1.6	21.4 ± 1.5	21.7 ± 1.7	> 0.05
GE_%LT2_ [%]	21.6 ± 1.5	21.5 ± 1.5	21.7 ± 1.6	> 0.05
Lactate threshold				
%${\dot{V}}\textrm{O}_{2_\textrm{peak}}$ at LT2 [%]	82.7 ± 4.0	83.2 (81.2-85.4)	80.7 (78.6-83.2)	> 0.05^w^
Power output at LT2 [W]	223 ± 49	235 ± 51	190 ± 25	< 0.001
bLa at LT2 [mmol⋅L^–1^]	3.51 ± 0.71	3.55 ± 0.72	3.41 ± 0.67	> 0.05
_mod_LT2_fix_ [W]	214 ± 45	224 ± 47	185 ± 23	< 0.001
bLa at _mod_LT2_fix_ [mmol⋅L^–1^]	3.02 ± 0.69	2.97 ± 0.70	3.14 ± 0.65	> 0.05
$_{\textrm{mod}}{\textrm{LT2}}_{\% {\dot{V}}\textrm{O}_{2_\textrm{peak}}}$ [W]	219 ± 50	230 ± 51	186 ± 26	< 0.001
bLa at $_{\textrm{mod}}{\textrm{LT2}}_{\% {\dot{V}}\textrm{O}_{2_\textrm{peak}}}$ [mmol⋅L^–1^]	3.33 ± 0.83	3.37 ± 0.84	3.23 ± 0.83	> 0.05
_mod_LT2_%LT2_ [W]	219 ± 50	231 ± 51	186 ± 25	< 0.001
bLa at _mod_LT2_%LT2_ [mmol⋅L^–1^]	3.36 ± 0.75	3.41 ± 0.76	3.24 ± 0.73	> 0.05

*p* values refer to the pairwise comparison between sexes with $^{\textrm{w}}$ indicating that Wilcoxon rank-sum test was used instead of *t*–test

*bLa*: blood lactate concentration, ${\textrm{Cc}}_{\textrm{fix}}$: Cc determined at 3 W⋅kg^–1^, ${\textrm{Cc}}_{\%{\dot{V}}\textrm{O}_{2_\textrm{peak}}}$: Cc determined at 75% of ${\dot{V}}\textrm{O}_{2_\textrm{peak}}$, ${\textrm{Cc}}_{\%{\rm LT}2}$: Cc determined at 90% of power output at LT2, GE_fix_: GE determined at 3 W⋅kg^–1^, ${\textrm{GE}}_{\%{\dot{V}}\textrm{O}_{2_\textrm{peak}}}$: GE determined at 75% of ${\dot{V}}\textrm{O}_{2_\textrm{peak}}$, GE_%LT2_: GE determined at 90% of power output at LT2, %${\dot{V}}\textrm{O}_{2_\textrm{peak}}$: fractional utilization of ${\dot{V}}\textrm{O}_{2_\textrm{peak}}$, LT2: lactate threshold 2, _mod_LT2_fix_: power output at LT2 modeled using ${\textrm{Cc}}_{\textrm{fix}}$, $_{\textrm{mod}}{\textrm{LT2}}_{\% {\dot{V}}\textrm{O}_{2_\textrm{peak}}}$: power output at LT2 modeled using ${\textrm{Cc}}_{\%{\dot{V}}\textrm{O}_{2_\textrm{peak}}}$, _mod_LT2_%LT2_: power output at LT2 modeled using ${\textrm{Cc}}_{\%{\rm LT}2}$

### Procedures

As part of a larger check-up in a local performance diagnostics center between April 2017 and June 2023, the athletes conducted an incremental step test on an SRM cycle ergometer (Schoberer Rad Meßtechnik SRM GmbH, Jülich, Germany) under constant laboratory conditions in terms of relative humidity (i.e., 35 ± 8%) and temperature (i.e., 20.5 ± 1.8$^{\circ }$C). After a 2-min resting measurement in a seated position on the ergometer, an initial resistance between 40 and 120 W was set depending on the athletes’ body mass (i.e., $\sim$1.5 W⋅kg^–1^), which was increased by 20 W every 3 min until volitional exhaustion (Zwingmann et al. 2019). For determination of bLa, a capillary blood sample of 20 $\upmu$L was taken from the earlobe in the last 30 s of each step and analyzed immediately after the test (Biosen C-line; EKF Diagnostic Sales, Magdeburg, Germany, coefficient of variation: ± 1.5%). Throughout the test, breathing gases (Metalyzer$\circledR$3B; Cortex Biophysik GmbH, Leipzig, Germany) and heart rate (Polar H7 Sensor, Polar Electro Oy, Kempele, Finnland or HRM-Swim™, Garmin Deutschland GmbH, Garching, Germany) were recorded every second. The spirometer was calibrated with a reference gas (5% CO_2_, 15% O_2_) every 2 weeks and before each test with ambient air and a 3-L syringe, according to the manufacturer’s specifications.

The subset of 36 triathletes (13 females and 23 males) conducted an additional 15-s all-out sprint test prior to the outlined step test. Following a 10-min warm-up at 2 W⋅kg^–1^, the sprint test was performed with athletes seated on the ergometer, adjusted to an isokinetic mode with a cadence of 120 rpm. Throughout the test, all athletes were verbally encouraged to achieve maximal power output. Capillary blood samples were taken before the test and during a 10-min passive resting phase from the second until the ninth minute afterward (every minute) to determine ${\dot{c}}La_\textrm{max}$ according to Eq. 2 (Quittmann et al. 2020; Heck et al. 2003). It relates the change in bLa levels, i.e., the highest bLa after (La_peak_) compared to before the sprint (La_rest_), to the respective exercise duration (t_exerc_) minus the theoretical alactic time (t_alac_), which was set to 3.5 s (Heck et al. 2003). After the passive rest ($\sim$10 min), athletes cycled at $\sim$2 W⋅kg^–1^ until baseline levels (i.e., $\le$ 1.5 mmol⋅L^–1^) were reached again.

2 $$\begin{aligned} {\dot{c}}La_\textrm{max} \, = \, \frac{\left( La_\textrm{peak}-La_\textrm{rest}\right) }{\left( t_\textrm{exerc}-t_\textrm{alac}\right) }. \end{aligned}$$

### Parameters

For the determination of power output at LT2, the measured bLa values were plotted against cycling power and fitted by a third-order polynomial function. The point on the polynomial fitting that yielded the maximal perpendicular distance to a straight line between the point of the first rise in bLa (slope = 0.01) and the last data point (i.e., the highest power output and bLa) was set to be LT2 as a valid estimate of MLSS (Zwingmann et al. 2019).

${\dot{V}}\textrm{O}_{2_\textrm{peak}}$ was defined as the highest 30-s moving average of ${\dot{V}}\textrm{O}_{2}$ in the test. Exhaustion was verified based on at least two of the following criteria (Midgley et al. 2007): respiratory exchange ratio $\ge$ 1.10, heart rate $\ge$ 95% of age predicted maximum, bLa $\ge$ 8 mmol⋅L^–1^, and volitional exhaustion. Data of three athletes who only reached one criterion (i.e., volitional exhaustion) were retained in the analysis as statistical evaluation confirmed that they had no significant impact on the overall results.

Using the equation of Støren et al. (2014), power output at LT2 (from now on referred to as _mod_LT2) was modeled using ${\dot{V}}\textrm{O}_{2_\textrm{peak}},$ %${\dot{V}}\textrm{O}_{2_\textrm{peak}}$ at LT2, and either of the three methods for calculating Cc (see below). All submaximal spirometric data (i.e., until LT2) were averaged over the last 60 s of each stage and a linear regression was applied to the averaged data to calculate the following variables. %${\dot{V}}\textrm{O}_{2_\textrm{peak}}$ at LT2 was calculated by dividing ${\dot{V}}\textrm{O}_{2}$ at LT2 by ${\dot{V}}\textrm{O}_{2_\textrm{peak}}.$ Cc and GE were determined at three different intensities: (1) a fixed power of 3 W⋅kg^–1^, which represents a midpoint between starting intensity and peak power output in the step test (see Table 1). (2) 75% of ${\dot{V}}\textrm{O}_{2_\textrm{peak}}$ to investigate a high percentage of ${\dot{V}}\textrm{O}_{2_\textrm{peak}}$ close to LT2, consistent with previous studies (Ji et al. 2023; Støren et al. 2013). (3) 90% of power output at LT2 representing a value close to maximal metabolic steady state without the risk of determining Cc above the true steady state (as the expected deviation of power at LT2 from power at MLSS was around 9% according to the validation study by Zwingmann et al. (2019)). Cc was calculated by dividing ${\dot{V}}\textrm{O}_{2}$ values by the three different work rates described above (i.e., ${\textrm{Cc}}_{\textrm{fix}}$, ${\textrm{Cc}}_{\%{\dot{V}}\textrm{O}_{2_\textrm{peak}}}$, and ${\textrm{Cc}}_{\%{\rm LT}2}$) and GE was calculated for each work rate taking into account the measured ${\dot{V}}\textrm{O}_{2}$ and carbon dioxide production according to Jeukendrup and Wallis (2005) (i.e., GE_fix_, ${\textrm{GE}}_{\%{\dot{V}}\textrm{O}_{2_\textrm{peak}}}$, and GE_%LT2_). These different Cc determination methods were used to calculate the three _mod_LT2 variants, i.e., _mod_LT2_fix_, $_{\textrm{mod}}{\textrm{LT2}}_{\% {\dot{V}}\textrm{O}_{2_\textrm{peak}}}$, and _mod_LT2_%LT2_.

### Statistical analysis

For statistical analysis, the statistics software R-Studio (version 4.1.3; Posit Software, PBC, Boston, United States) was used. Data are presented as mean ± standard deviation (SD) if normal distribution was assumed, and as median (interquartile range) if not. An alpha level of 0.05 was applied for all statistical tests. All analyses were performed for the whole data set and separately for male and female subgroups. Prior to inferential statistics, normal distribution and homogeneity of variance were visually verified using Q-Q and residual plots. Further, for the multiple regression analyses, residuals were checked in the same way.

To investigate statistical sex differences, independent sample *t*-tests were used. A Wilcoxon rank-sum test was carried out for individual variables for which no normal distribution was assumed as indicated by a ^w^ in the Results section. To investigate the physiological model accuracy, a Bland-Altman analysis was performed, with mean difference (i.e., fixed bias) tested using a *t*-test for statistical deviation of 0, limits of agreement calculated as 1.96-fold SD (i.e., random bias), and proportional bias examined by significance testing of the slope of the regression line between the differences and the means. In addition, intra-class correlation coefficients (ICC) were determined in two-way mixed model single-measurements with absolute agreement for power output at modeled and experimentally determined LT2 based on the different calculation methods for Cc. The degree of agreement was interpreted: < 0.50 = poor, ${0.50}-{0.75}$ = moderate, ${0.75}-{0.90}$ = good, and > 0.90 = excellent (Koo and Li 2016).

Further, the association between the physiological model predictors (${\dot{V}}\textrm{O}_{2_\textrm{peak}},$ %${\dot{V}}\textrm{O}_{2_\textrm{peak}}$ at LT2, and Cc) and the criterion (power output at LT2) was assessed with bivariate correlation and multiple regression analyses using Pearson’s *r* and the bi-directional stepwise selection procedure (criterion: reduction in Akaike information criterion), respectively. In addition, in the mixed subgroup, which had also performed the sprint test as described above, it was tested whether incorporating ${\dot{c}}La_\textrm{max}$ as an additional predictor improved the regression. To further quantify and explain the unique and common contribution of the predictors to power output at LT2, commonality analysis was conducted in accordance with previous work (Ji et al. 2023; Ray-Mukherjee et al. 2014). Lastly, to assess the influence of age on the physiological model, it was correlated with the physiological model predictors, the experimentally determined and modeled power output at LT2, and the difference between them using Spearman’s $\rho$ (due to the deviation from normal distribution in age). The following interpretation was used for both Pearson’s *r* and Spearman’s $\rho$: < 0.30 = negligible, ${0.30}-{0.50}$ = low, ${0.50}-{0.70}$ = moderate, ${0.70}-{0.90}$ = high, and > 0.90 = very high (Mukaka 2012).

## Results

The anthropometric and physiological characteristics of the participants along with sex differences are summarized in Table 1. For a better understanding of the physiological modeling procedure, the following example demonstrates the calculation of power output at LT2 according to Eq. 1. For a representative cyclist with a ${\dot{V}}\textrm{O}_{2_\textrm{peak}}$ of 3456 mL⋅min^–1^, a %${\dot{V}}\textrm{O}_{2_\textrm{peak}}$ of 85%, and a ${\textrm{Cc}}_{\%{\rm LT}2}$ of 13.6 mL⋅min^–1^⋅W^–1^ the following modeled power at LT2 results:

$$\begin{aligned} \text {Power output at LT2} = (85\%) \cdot \frac{3456 \, \text {mL} \cdot \text {min}^{-1}}{13.6 \, \text {mL} \cdot \text {min}^{-1} \cdot \text {W}^{-1}} = 216 \, \text {W}. \end{aligned}$$

**Fig. 1:**
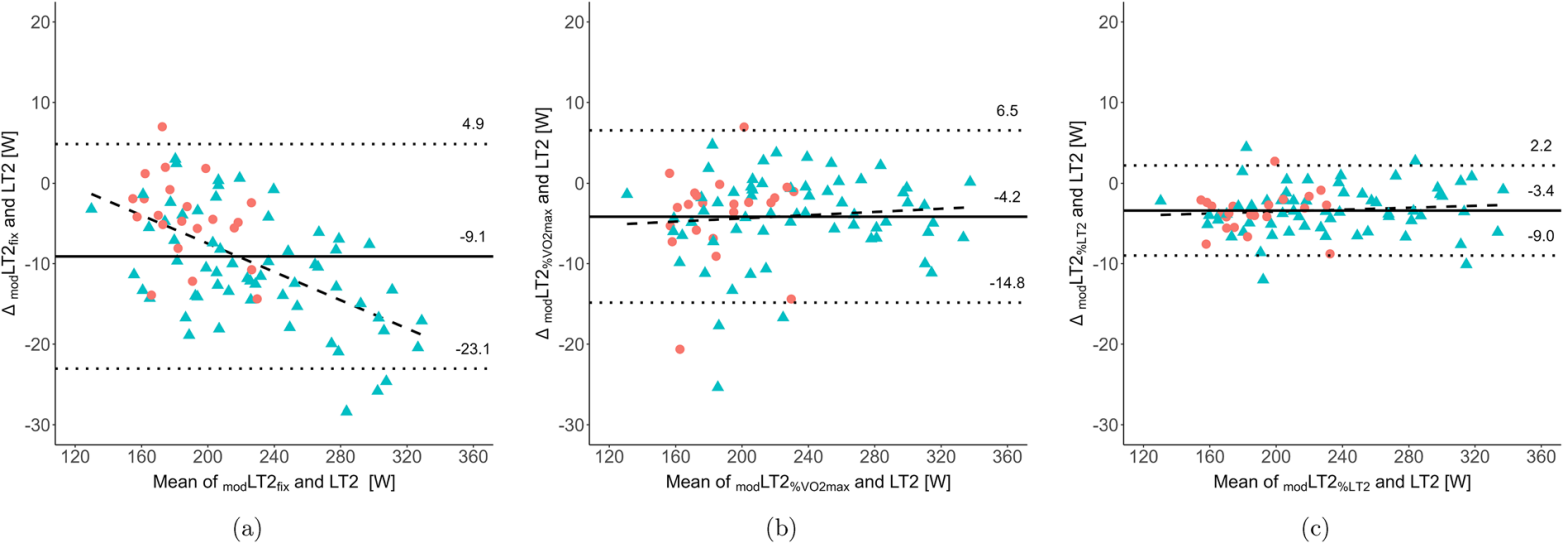
Bland-Altman plots showing the absolute differences between modeled power output at lactate threshold 2 using oxygen cost of cycling (**a**) at a fixed power of 3 W⋅kg^–1^ (_mod_LT2_fix_), (**b**) at 75% of maximal oxygen uptake ($_{\textrm{mod}}{\textrm{LT2}}_{\% {\dot{V}}\textrm{O}_{2_\textrm{peak}}}$), and (**c**) at 90% of lactate threshold 2 (_mod_LT2_%LT2_) vs. power output at lactate threshold 2 (LT2) determined by modified maximal deviation method. The individual data of male (*N* = 61) and female (*N* = 22) athletes are presented by blue triangles and red circles, respectively. The solid line indicates mean difference (fixed bias), the dotted lines mark the limits of agreement (mean ± 1.96-fold standard deviation; random bias), and the dashed line represents the fitted linear regression (proportional bias)

The fixed and random biases between _mod_LT2_fix_, $_{\textrm{mod}}{\textrm{LT2}}_{\% {\dot{V}}\textrm{O}_{2_\textrm{peak}}}$, and _mod_LT2_%LT2_ vs. power output at LT2 are illustrated in the Bland-Altman plots (Fig. 1, showing absolute values [W]) and listed in Table 2 (showing relative values [%]). In addition, Table 2 presents ICC results with the highest values for _mod_LT2_%LT2_ for all, male, and female athletes (0.996, 0.996, and 0.986, respectively). Therefore, this threshold model was used for all further analyses.

**Table 2:** Mean percentage difference (± limits of agreement) and intra-class correlation coefficients (ICC, along with confidence intervals [CI]) between modeled power output at lactate threshold 2 using oxygen cost of cycling at a fixed power of 3 W⋅kg^–1^ (_mod_LT2_fix_), at 75% of maximal oxygen uptake ($_{\textrm{mod}}{\textrm{LT2}}_{\% {\dot{V}}\textrm{O}_{2_\textrm{peak}}}$), and at 90% of lactate threshold 2 (_mod_LT2_%LT2_) vs. experimentally determined power output at lactate threshold 2

	Mean difference [%]	Fixed bias *p*–value	Proportional bias regression line (*p–*value)	ICC (95% CI)
**All** (*N* = 83)				
_mod_LT2_fix_	$-4.2$ ± 6.4	0.22	*y* = − 0.000*x* + 0.008 (< 0.001)	0.971 (${0.607}-{0.991}$)
$_{\textrm{mod}}{\textrm{LT2}}_{\% {\dot{V}}\textrm{O}_{2_\textrm{peak}}}$	$-1.9$ ± 4.8	0.59	*y* = 0.000*x* − 0.044 (> 0.05)	0.991 (${0.961}-{0.996}$)
_mod_LT2_%LT2_	$-1.5$ ± 2.5	0.66	*y* = 0.000*x* − 0.036 (< 0.01)	0.996 (${0.942}-{0.999}$)
**Males** (*N* = 61)				
_mod_LT2_fix_	$-4.7$ ± 6.0	0.22	*y* = − 0.000*x* − 0.014 (> 0.05)	0.966 (${0.367}-{0.991}$)
$_{\textrm{mod}}{\textrm{LT2}}_{\% {\dot{V}}\textrm{O}_{2_\textrm{peak}}}$	$-1.8$ ± 4.6	0.65	*y* = 0.000*x* − 0.047 (> 0.05)	0.991 (${0.960}-{0.996}$)
_mod_LT2_%LT2_	$-1.4$ ± 2.5	0.72	y = 0.000*x* − 0.034 (< 0.05)	0.996 (${0.955}-{0.999}$)
**Females** (*N* = 22)				
_mod_LT2_fix_	$-2.3$ ± 5.5	0.55	*y* = − 0.000*x* + 0.049 (> 0.05)	0.961 (${0.797}-{0.987}$)
$_{\textrm{mod}}{\textrm{LT2}}_{\% {\dot{V}}\textrm{O}_{2_\textrm{peak}}}$	$-2.1$ ± 5.8	0.61	*y* = 0.000*x* − 0.054 (> 0.05)	0.965 (${0.861}-{0.988}$)
_mod_LT2_%LT2_	$-1.9$ ± 2.5	0.64	*y* = 0.000*x* − 0.049 (> 0.05)	0.986 (${0.630}-{0.997}$)

Combining ${\dot{V}}\textrm{O}_{2_\textrm{peak}}$, %${\dot{V}}\textrm{O}_{2_\textrm{peak}}$ at LT2, and ${\textrm{Cc}}_{\%{\rm LT}2}$ explained 99%, 99%, and 98% of the variance in power output at LT2 for all, male, and female athletes, respectively (Table 3). Examining the correlations between the model predictors and power output at LT2, ${\dot{V}}\textrm{O}_{2_\textrm{peak}}$ (*r* > 0.75,* p* < 0.001) and ${\textrm{Cc}}_{\%{\rm LT}2}$ (*r* < $-0.{47}$,* p* < 0.01) were significantly correlated with power output at LT2 for all, male, and female athletes. Conversely, %${\dot{V}}\textrm{O}_{2_\textrm{peak}}$ at LT2 was not significantly correlated with power output at LT2 (0.07 <* r* < 0.14,* p* > 0.13) (Table 3).

**Table 3:** Summary of stepwise multiple regression analyses using power output at lactate threshold 2 (LT2) [W] as dependent variable and the classical physiological parameters as independent variables

Model	Variable	Beta	SE	Std. Beta	*t*	*p*	*r*	${{R^{2}}}$	${{Adj. \,R^{2}}}$	*F*	*p*	AIC
**Analysis 1: All (*N* = 83)**												
1	(Constant)	$-1{2.50}$	10.96		$-{1.14}$	> 0.05		0.855	0.853	478	< 0.001	490
	${\dot{V}}\textrm{O}_{2_\textrm{peak}}$	0.07	< 0.01	0.92	21.85	< 0.001						
2	(Constant)	215.00	21.87		9.83	< 0.001		0.942	0.941	651	< 0.001	415
	${\dot{V}}\textrm{O}_{2_\textrm{peak}}$	0.06	< 0.01	0.86	31.02	< 0.001						
	${\textrm{Cc}}_{\%{\rm LT}2}$	$-1{5.83}$	1.44	$-0.3{0}$	$-1{0.97}$	< 0.001						
3	(Constant)	$-1{1.95}$	14.42		$-0.8{3}$	> 0.05		0.991	0.990	2742	< 0.001	268
	${\dot{V}}\textrm{O}_{2_\textrm{peak}}$	0.06	< 0.01	0.87	77.30	< 0.001	0.925					
	${\textrm{Cc}}_{\%{\rm LT}2}$	$-16.00$	0.59	$-0.31$	$-27.{19}$	< 0.001	$-0.4{96}$					
	%${\dot{V}}\textrm{O}_{2_\textrm{peak}}$at LT2	2.72	0.14	0.22	20.04	> 0.05	0.143					
**Analysis 2: Males(** ***N*** **= 61)**												
1	(Constant)	$-1{8.82}$	14.01		$-1.{34}$	> 0.05		0.852	0.849	338	< 0.001	365
	${\dot{V}}\textrm{O}_{2_\textrm{peak}}$	0.07	< 0.01	0.92	18.39	< 0.001						
2	(Constant)	241.98	29.08		8.32	< 0.001		0.941	0.939	465	< 0.001	311
	${\dot{V}}\textrm{O}_{2_\textrm{peak}}$	0.06	< 0.01	0.81	23.84	< 0.001						
	${\textrm{Cc}}_{\%{\rm LT}2}$	$-17.{2}6$	1.83	$-0.3{2}$	$-9.{42}$	< 0.001						
3	(Constant)	$-2{1.04}$	19.78		$-1.{06}$	> 0.05		0.990	0.990	1900	< 0.001	204
	${\dot{V}}\textrm{O}_{2_\textrm{peak}}$	0.06	< 0.01	0.85	59.63	< 0.001	0.923					
	${\textrm{Cc}}_{\%{\rm LT}2}$	$-16.3{1}$	0.76	$-0.30$	$-21.4{2}$	< 0.001	$-0.605$					
	%${\dot{V}}\textrm{O}_{2_\textrm{peak}}$at LT2	2.87	0.17	0.22	17.11	< 0.001	0.088					
**Analysis 3: Females (*N* = 22)**												
1	(Constant)	26.31	32.08		0.82	> 0.05		0.568	0.546	26	< 0.001	126
	${\dot{V}}\textrm{O}_{2_\textrm{peak}}$	0.06	0.01	0.75	5.12	< 0.001						
2	(Constant)	211.37	34.44		6.14	0.001		0.862	0.848	60	< 0.001	103
	${\dot{V}}\textrm{O}_{2_\textrm{peak}}$	0.05	< 0.01	0.74	8.72	< 0.001						
	${\textrm{Cc}}_{\%{\rm LT}2}$	$-13.88$	2.18	$-0.54$	$-6.38$	< 0.001						
3	(Constant)	19.54	22.72		0.86	> 0.05		0.981	0.977	301	< 0.001	62
	${\dot{V}}\textrm{O}_{2_\textrm{peak}}$	0.06	< 0.01	0.84	24.51	< 0.001	0.753					
	${\textrm{Cc}}_{\%{\rm LT}2}$	$-14.80$	0.85	$-0.58$	$-17.47$	< 0.001	$-0.558$					
	%${\dot{V}}\textrm{O}_{2_\textrm{peak}}$at LT2	2.24	0.22	0.36	10.43	< 0.001	0.071					

*AIC*: Akaike information criterion, *Beta*: beta coefficient, *SE*: standard error, *Std. Beta*: standardized beta coefficient, *r*: bivariate Pearson correlation with power at lactate threshold 2, $R^2$: coefficient of determination, *Adj. *$R^2$: adjusted coefficient of determination, ${\dot{V}}\textrm{O}_{2_\textrm{peak}}$: maximal oxygen uptake [mL⋅min^–1^], ${\textrm{Cc}}_{\%{\rm LT}2}$: oxygen cost of cycling determined at 90% of LT2 [mL⋅min^–1^⋅W^–1^], %${\dot{V}}\textrm{O}_{2_\textrm{peak}}$: fractional utilization of ${\dot{V}}\textrm{O}_{2_\textrm{peak}}$ [%]

**Fig. 2:**
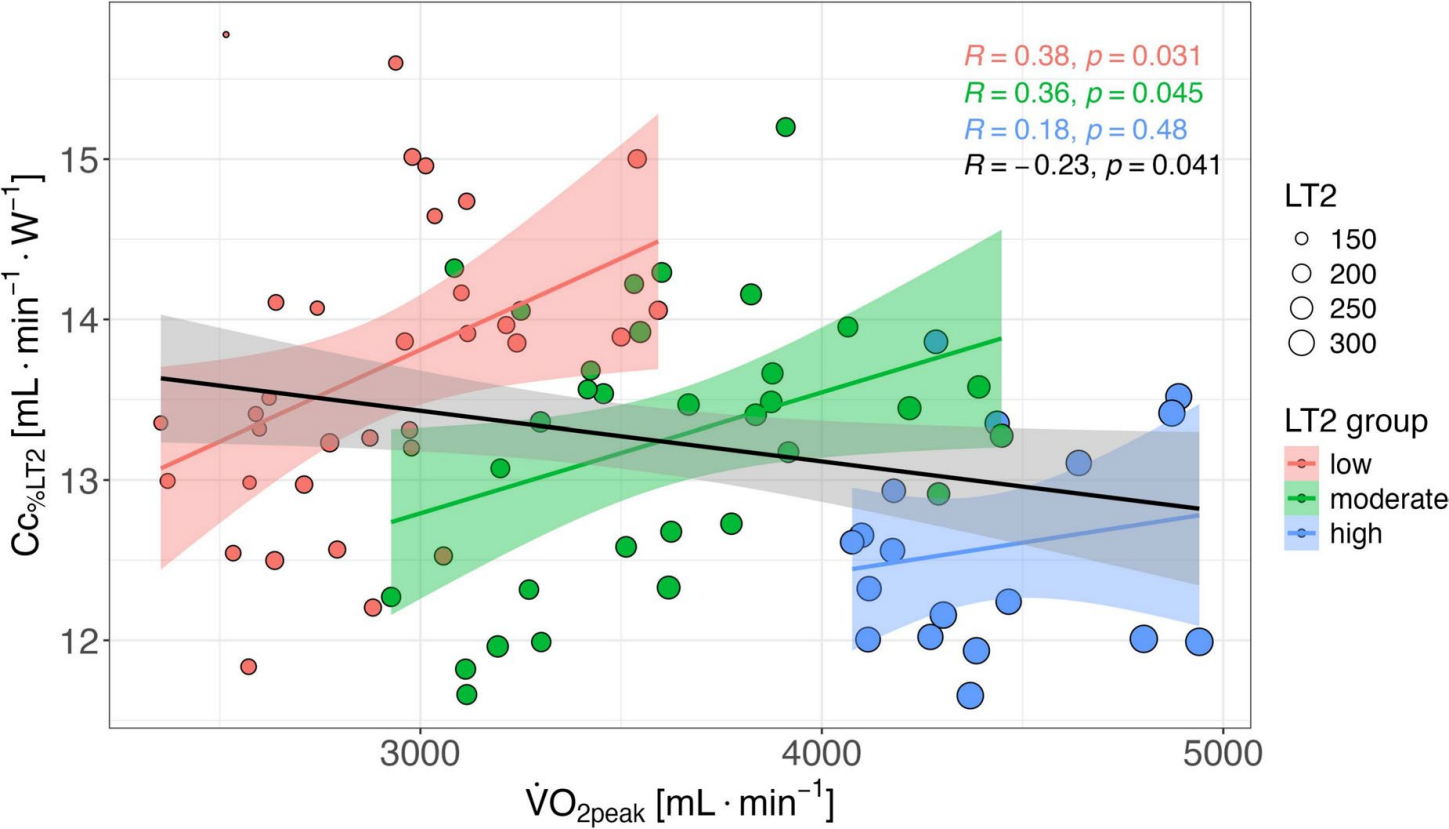
Scatter plot illustrating the correlation between maximal oxygen uptake (${\dot{V}}\textrm{O}_{2_\textrm{peak}}$) and oxygen cost of cycling (${\textrm{Cc}}_{\%{\rm LT}2}$). The straight black line represents the overall correlation for the entire sample, whereas the three subgroups, distinguished by power output at lactate threshold 2 (LT2; i.e., the range was divided into three equal intervals), are depicted by different colored arrays. Within these subgroups, the colored straight lines again indicate the correlation between ${\dot{V}}\textrm{O}_{2_\textrm{peak}}$ and ${\textrm{Cc}}_{\%{\rm LT}2}$. The size of the dots reflects the performance level based on power output at LT2

Besides the correlation between model predictors and power output at LT2, Fig. 2 depicts the correlation between the most important model predictors (i.e., ${\dot{V}}\textrm{O}_{2_\textrm{peak}}$ and Cc) across the whole sample and grouped according to power output at LT2 (i.e., three groups with equal intervals based on the range). Further, ${\dot{c}}La_\textrm{max}$ was 0.48 ± 0.10 mmol⋅L^–1^⋅s^–1^ in the subgroup of 36 triathletes and not significantly different between sexes (*p* $=$ 0.34). In this subgroup, ${\dot{c}}La_\textrm{max}$ did not correlate statistically with power output at LT2 (*r* = 0.23, *p* = 0.18), nor did it contribute significantly to explaining variance in the multiple regression analysis in addition to or instead of the variables ${\dot{V}}\textrm{O}_{2_\textrm{peak}},$ %${\dot{V}}\textrm{O}_{2_\textrm{peak}}$ at LT2 and ${\textrm{Cc}}_{\%{\rm LT}2}$. Further, ${\dot{c}}La_\textrm{max}$ did not show any statistically significant correlation with either of the other model predictors ($-0.10$ < *r* < 0.16, *p* > 0.36) except for ${\textrm{Cc}}_{\%{\rm LT}2}$ (*r* = $-0.41$, *p* = 0.01).

**Fig. 3:**
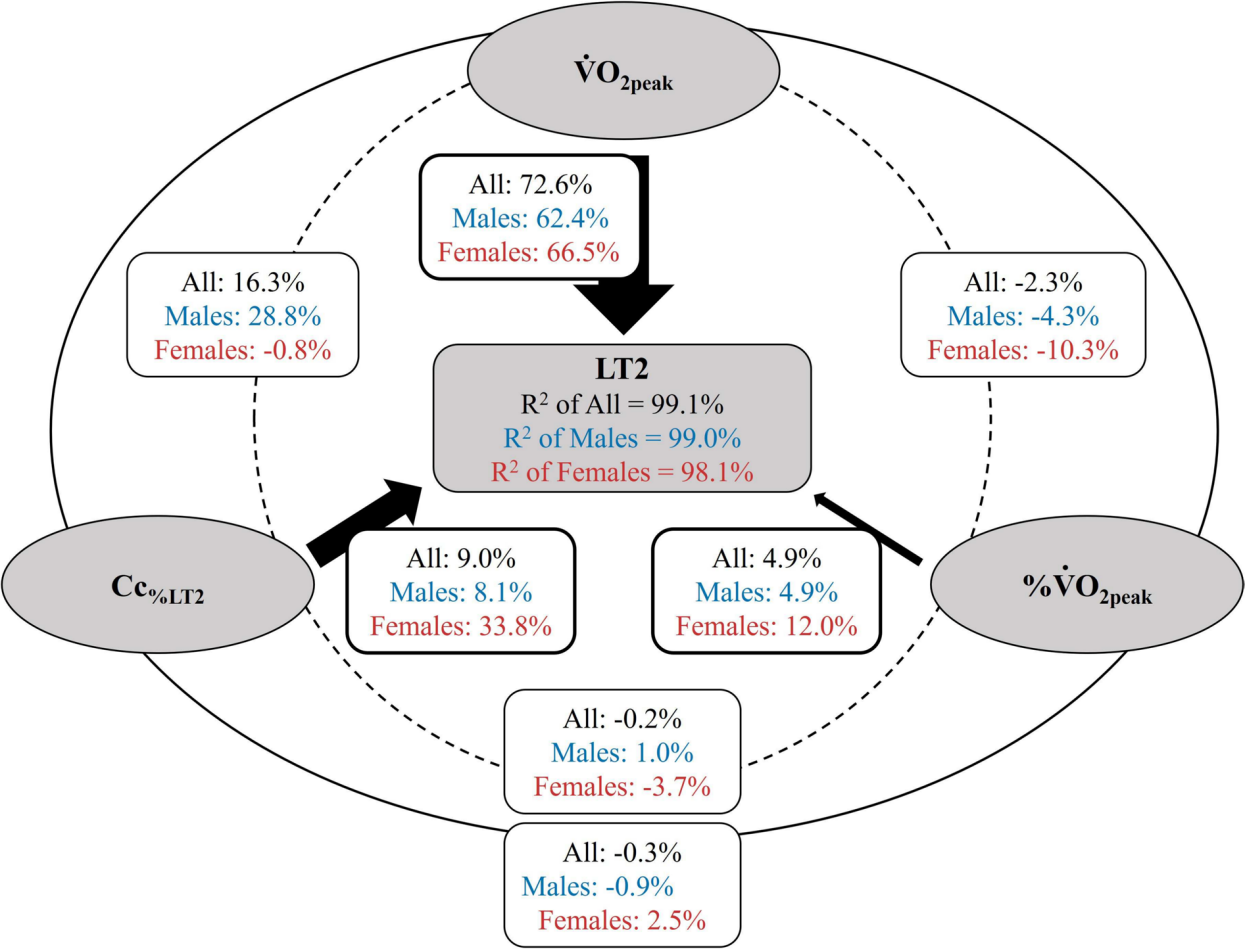
Graphical summary of commonality analyses for the experimentally determined power output at lactate threshold 2 (LT2) across all (*N* = 83), male (*N* = 61), and female (*N* = 22) athletes. The percentage contribution of each unique predictor to the total regression $R^2$ is presented by the black-filled arrows; the dashed lines and external solid lines represent the common effects of two and all predictors in $R^2$, respectively. ${\dot{V}}\textrm{O}_{2_\textrm{peak}}$: maximal oxygen uptake, ${\textrm{Cc}}_{\%{\rm LT}2}$: oxygen cost of cycling determined at 90% of LT2, %${\dot{V}}\textrm{O}_{2_\textrm{peak}}$: fractional utilization of ${\dot{V}}\textrm{O}_{2_\textrm{peak}}$ at LT2

Consistent with the multiple regression analyses, commonality analysis revealed the highest contribution of ${\dot{V}}\textrm{O}_{2_\textrm{peak}}$ to the total regression $R^2$ for all, male, and female athletes (> 62%). While ${\textrm{Cc}}_{\%{\rm LT}2}$ and %${\dot{V}}\textrm{O}_{2_\textrm{peak}}$ at LT2 only showed a small contribution to power output at LT2 in all and in male athletes (< 9% and < 5%, respectively), the contribution was greater in female athletes (34% and 12%, respectively). The most apparent common effect was detected between ${\dot{V}}\textrm{O}_{2_\textrm{peak}}$ and ${\textrm{Cc}}_{\%{\rm LT}2}$ in all and in male athletes (16% and 29%, respectively), which was negligible in female athletes ($-1$%). Comprehensive results of the commonality analyses are shown in Fig. 3.

**Fig. 4:**
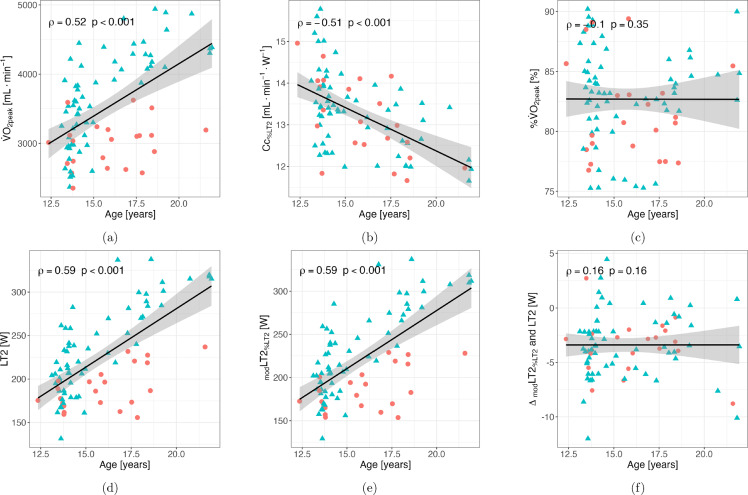
Scatter plots showing the Spearman’s rank correlation *ρ* between age and (**a**) maximal oxygen uptake (${\dot{V}}\text{O}_{2_\textrm{peak}}$), (**b**) Oxygen cost of cycling determined at 90% of power output at lactate threshold 2 (${\text{Cc}}_{\%{\rm LT}2}$), (**c**) fractional utilization of ${\dot{V}}\text{O}_{2_\textrm{peak}}$ at lactate threshold 2, (**d**) power output at lactate threshold 2 (LT2) determined using the modified maximal deviation method, (**e**) modeled power output at LT2 using oxygen cost of cycling at 90% of power output at LT2, and (**f**) the difference between modeled and experimentally determined power output at LT2. Individual data of male (*N* = 61) and female (*N* = 22) athletes are presented by blue triangles and red circles, respectively. The straight black line represents the linear regression

The correlations between age and the physiological model predictors, the experimentally determined and modeled power output at LT2, and the difference between them are depicted in Fig. 4.

## Discussion

The aim of the study was to test the accuracy of modeling power output at LT2 based on the physiological variables ${\dot{V}}\textrm{O}_{2_\textrm{peak}}$, %${\dot{V}}\textrm{O}_{2_\textrm{peak}}$ at LT2, and Cc using different determination methods in cycling in young athletes depending on sex and to investigate the influence of the model predictors and ${\dot{c}}La_\textrm{max}$ on power at LT2. Excellent agreement was found between the power outputs at modeled LT2 and the LT2 criterion determined from bLa kinetics, regardless of sex and across different determination methods for Cc, supporting the suitability of the model also in young and female athletes in cycling. Using ${\textrm{Cc}}_{\%{\rm LT}2}$ resulted in the highest accordance between _mod_LT2 and power output at LT2 in all, male, and female athletes, respectively. In both sexes, ${\dot{V}}\textrm{O}_{2_\textrm{peak}}$ demonstrated the largest impact on power output at LT2, followed by Cc, which was more important for female athletes, while %${\dot{V}}\textrm{O}_{2_\textrm{peak}}$ at LT2 only had a negligible influence. In contrast, ${\dot{c}}La_\textrm{max}$ did not contribute significantly to power output at LT2.

The multiple regression analyses revealed that 99% of the total variance could be explained by the three physiological variables ${\dot{V}}\textrm{O}_{2_\textrm{peak}}$, %${\dot{V}}\textrm{O}_{2_\textrm{peak}}$ at LT2, and ${\textrm{Cc}}_{\%{\rm LT}2}$ in all and the male athletes and 98% in the females. This almost perfect explanation of variance underlines the importance of the three parameters for endurance performance and is in accordance with high accuracy demonstrated for estimating work rate at LT2 using _mod_LT2 in other sports, e.g., 98% in male and female adult cross-country skiers (Johansen et al. 2022), 90% in adult runners of both sexes (Støa et al. 2020), or 95% in male adult cyclists (Støren et al. 2014). However, the previous studies used LT2 defined as the power at warm-up value +2.3 mmol⋅L^–1^ as the criterion. Due to the lack of validation against MLSS and the susceptibility of fixed bLa values to external confounders such as test protocol characteristics, training, and nutrition status, a validated threshold concept based on bLa kinetics (i.e., Zwingmann et al. 2019; Jamnick et al. 2018) was used in the present study. In accordance with previous work (Ji et al. 2023), this approach allowed even a higher variance explanation compared to the fixed threshold concept (i.e., Støa et al. 2020; Støren et al. 2014), likewise highlighting the applicability of the model to young endurance athletes in cycling.

Similar to a previous study in running (Ji et al. 2023), different assessment methods for Cc were evaluated to optimize the agreement of _mod_LT2 and the experimentally determined power output at LT2. Despite high similarity between the three Cc determination methods, differences in the accuracy of modeling power output at LT2 were detected. Specifically, incorporating ${\textrm{Cc}}_{\%{\rm LT}2}$ into the physiological model (i.e., _mod_LT2_%LT2_) showed superior accuracy in modeling power at LT2 with the highest ICC values along with the lowest fixed and random bias. In accordance with the assumption that determination of Cc at a work rate that closely mirrors the demands of the target intensity (e.g., movement mechanics in competition) would be also better suited for performance prediction (Lacour and Bourdin 2015; Ji et al. 2023), ${\textrm{Cc}}_{\%{\rm LT}2}$ was superior to ${\textrm{Cc}}_{\textrm{fix}}$. Thus, the use of a fixed power output of 3 W⋅kg^–1^ (i.e., ${\textrm{Cc}}_{\textrm{fix}}$) resulted in assessing Cc at a lower work rate, both in absolute (177 vs. 199 vs. 200 W) and relative terms (69 vs. 75 vs. 76% of ${\dot{V}}\textrm{O}_{2_\textrm{peak}}$), compared to ${\textrm{Cc}}_{\%{\dot{V}}\textrm{O}_{2_\textrm{peak}}}$ and ${\textrm{Cc}}_{\%{\rm LT}2}$. Besides the lower work rate, assessing Cc at a fixed power output elicited heterogeneous metabolic responses (e.g., substrate utilization and percent utilization of ${\dot{V}}\textrm{O}_{2_\textrm{peak}}$ [57–91%]), leading to inter-individual variability and consequently inferior estimation of power at LT2. In addition, it is noteworthy that despite assessing Cc at the same relative intensity with respect to ${\dot{V}}\textrm{O}_{2_\textrm{peak}}$ (${\textrm{Cc}}_{\%{\dot{V}}\textrm{O}_{2_\textrm{peak}}}$), _mod_LT2_%LT2_ still exhibited a lower random bias in prediction of power output at LT2 compared to $_{\textrm{mod}}{\textrm{LT2}}_{\% {\dot{V}}\textrm{O}_{2_\textrm{peak}}}$. Since utilizing ${\textrm{Cc}}_{\%{\rm LT}2}$ can reduce the inter-individual variability in acute metabolic responses to exercise (Meyler et al. 2023; Sabater-Pastor et al. 2022) and minimize the risk of occasionally using non-steady state ${\dot{V}}\textrm{O}_{2}$ values (i.e., above maximal metabolic steady state) (Mattioni Maturana et al. 2016), this potentially contributed to the higher accuracy of _mod_LT2_%LT2_ compared to $_{\textrm{mod}}{\textrm{LT2}}_{\% {\dot{V}}\textrm{O}_{2_\textrm{peak}}}$ (Scharhag-Rosenberger et al. 2010). Therefore, our findings strongly support the measurement of Cc relative to a bioenergetic threshold, ideally close to competition demands (e.g., at 90% of LT2).

Of the three variables, ${\dot{V}}\textrm{O}_{2_\textrm{peak}}$ correlated most strongly with power output at LT2 in all, male, and female athletes. Noticeably, the correlation was more pronounced in male compared to female athletes (*r* = 0.93 vs. *r* = 0.75). While the importance of a highly developed ${\dot{V}}\textrm{O}_{2_\textrm{peak}}$ for endurance performance (Ingjer 1991; Ingham et al. 2002) and its close relationship to power at LT2 has been frequently shown before (Johansen et al. 2022; Støren et al. 2014; Støa et al. 2020), lower correlations for female athletes could be at least partly related to the greater heterogeneity for ${\dot{V}}\textrm{O}_{2_\textrm{peak}}$ in males (range: 50–74 mL ⋅kg^–1^⋅min^–1^) compared to females (45–64 mL⋅kg^–1^⋅min^–1^). Interestingly, however, the range of ${\textrm{Cc}}_{\%{\rm LT}2}$ (${11.7}-{15.8}$ and ${11.7}-{15.0}$ mL⋅min^–1^⋅W^–1^ for males and females, respectively) and the correlation with power output at LT2 were similar for both sexes (*r* = $-0.6{1}$ and *r* = $-0.56$). Yet, in contrast to similarly high unique effects for ${\dot{V}}\textrm{O}_{2_\textrm{peak}}$ (i.e., 62 and 67% in male and female athletes), the commonality analyses revealed a much higher relevance of Cc in female (33%) compared to male athletes (8%). This is consistent with previous studies in young and adult runners that have shown a greater importance of C in females (Støa et al. 2020; Ji et al. 2023), which could be related to sex-specific differences in substrate metabolism (i.e., higher reliance on fat oxidation) or muscle tissue properties (i.e., greater proportion of type I fibers and capillarization) (Besson et al. 2022) or even lower trainability of ${\dot{V}}\textrm{O}_{2_\textrm{peak}}$ (Diaz-Canestro and Montero 2019).

Despite an additional 5% of variance explanation in the whole sample, %${\dot{V}}\textrm{O}_{2_\textrm{peak}}$ at LT2 did not correlate with power output at LT2 in young male (*r* = 0.09) and female athletes (*r* = 0.07), underlining the minor importance of this physiological variable for endurance performance in line with previous studies (Støa et al. 2010; Niemeyer et al. 2022; Støren et al. 2013; Ji et al. 2023), especially when compared to ${\dot{V}}\textrm{O}_{2_\textrm{peak}}$ and Cc. This is also highlighted by the non-significant correlation between age and %${\dot{V}}\textrm{O}_{2_\textrm{peak}}$ at LT2 in contrast to the statistically significant moderate positive correlation with ${\dot{V}}\textrm{O}_{2_\textrm{peak}}$ and power output at LT2 or _mod_LT2_%LT2_ and negative correlation with ${\textrm{Cc}}_{\%{\rm LT}2}$. Despite the cross-sectional nature of the data presented, these correlations indicate that older athletes show higher endurance performance based on higher ${\dot{V}}\textrm{O}_{2_\textrm{peak}}$ and lower ${\textrm{Cc}}_{\%{\rm LT}2}$, likely due to a combination of growth, maturation, and training experience (Marín-Pagán et al. 2021; Hopker et al. 2009), but not improved %${\dot{V}}\textrm{O}_{2_\textrm{peak}}$. Furthermore, the non-significant correlation between age and the difference between modeled and experimentally determined power at LT2 underscores the broad applicability and accuracy of the model across the age range of 12–21 years.

Further, in a subset of 36 athletes, ${\dot{c}}La_\textrm{max}$ neither correlated significantly with power output at LT2 nor did it explain additional variance in the multiple regression analysis. Despite some recent debates on the influence of ${\dot{c}}La_\textrm{max}$ as a marker of glycolytic power on endurance performance (e.g., Quittmann et al. 2023; Schünemann et al. 2023; Wackerhage et al. 2022), ${\dot{c}}La_\textrm{max}$ added no further information in our study, which is similar to the only 4% additional variance explanation besides ${\dot{V}}\textrm{O}_{2_\textrm{peak}}$, %${\dot{V}}\textrm{O}_{2_\textrm{peak}}$, and C for a 5-km run in the study by Quittmann et al. (2023). In addition, there was also no statistical association between %${\dot{V}}\textrm{O}_{2_\textrm{peak}}$ and ${\dot{c}}La_\textrm{max}$ (r = $-0.10$ for all athletes in the subset) contrary to theoretical assumptions (Wackerhage et al. 2022). Nevertheless, it can be speculated that the slight underestimation of power output at LT2 (i.e., 1.4–4.2% on average) by all _mod_LT2 variants might be due to a slight underestimation of total metabolic power input, as this only included the aerobic component (${\dot{V}}\textrm{O}_{2_\textrm{peak}}$) and not the anaerobic one, as previously suggested (Støren et al. 2013).

In addition to the cited unique effects, commonality analysis revealed a positive common effect between ${\dot{V}}\textrm{O}_{2_\textrm{peak}}$ and ${\textrm{Cc}}_{\%{\rm LT}2}$ in the male (29%), but not in the female athletes ($-1$%), indicating an additional variance explanation by the combination of both determinants. Since the coefficients did not change when GE_%LT2_ was used instead of ${\textrm{Cc}}_{\%{\rm LT}2}$, potentially expected differences in substrate utilization between sexes did not account for the different common effects (Besson et al. 2022). In general, conflicting findings have been reported on the association of ${\dot{V}}\textrm{O}_{2_\textrm{peak}}$ and C across various sports, e.g., a significant positive relationship in elite male adult cyclists (Lucía et al. 2002) as opposed to a significant negative correlation in sedentary women during walking (Hunter et al. 2005). Since Borgen (2018) argued that the positive relationship between ${\dot{V}}\textrm{O}_{2_\textrm{peak}}$ and C is mainly due to an endogenous selection bias as a result of often homogeneous (and high) level, we examined the relationship between the two variables across the whole sample and in three subgroups of more homogeneous performance levels (according to power output at LT2). In line with the assumptions of Borgen (2018), we observed a negative correlation between ${\dot{V}}\textrm{O}_{2_\textrm{peak}}$ and ${\textrm{Cc}}_{\%{\rm LT}2}$ when considering the whole sample and thus a very heterogeneous group, while an opposite positive correlation was found for the three subgroups with more homogeneous levels. This suggests that current endurance performance (as indicated by power at LT2) can be achieved by different combinations of ${\dot{V}}\textrm{O}_{2_\textrm{peak}}$ and Cc, but that both parameters need to be improved over time to reach a high performance level.

The following aspects should be considered when interpreting the study results. First, ${\dot{V}}\textrm{O}_{2_\textrm{peak}}$ may have been underestimated using an incremental step test instead of a shorter ramp test. However, despite the potential underestimation, this does not affect the model accuracy, as ${\dot{V}}\textrm{O}_{2_\textrm{peak}}$ appears in both the numerator (see Eq. 1) and denominator (as %${\dot{V}}\textrm{O}_{2_\textrm{peak}}$ is calculated by dividing ${\dot{V}}\textrm{O}_{2}$ at LT2 by ${\dot{V}}\textrm{O}_{2_\textrm{peak}}$) of the physiological model calculation. Second, we used a step duration of 3 min in the incremental test, which may not be sufficient for ${\dot{V}}\textrm{O}_{2}$ slow component expression (Iannetta et al. 2021). However, since the influence of the ${\dot{V}}\textrm{O}_{2}$ slow component on ${\dot{V}}\textrm{O}_{2}$ values below LT2 is considered marginal (Clark and Draper 2019) and Cc was only assessed at 90% LT2 in the present study, inaccurate determination of Cc is unlikely. Third, the criterion, i.e., power output at LT2, is not mathematically independent of the physiological model predictor ${\textrm{Cc}}_{\%{\rm LT}2}$. However, since the model accuracy changes only slightly when using the other two Cc determination methods or even peak power output from the incremental test as an alternative criterion, the influence of mathematical dependence seems minor. Lastly, an unequal number of male and female athletes were included in the study, reducing the power of inference statistics in the female compared to the male subgroup.

As the modeled power at LT2 corresponds closely to the experimentally determined values in young athletes in cycling, relevant training implications can be derived from the evaluation of the main determinants, i.e., ${\dot{V}}\textrm{O}_{2_\textrm{peak}}$ and Cc. Thus, training prescriptions can be tailored to the athletes, focusing either on the improvement of ${\dot{V}}\textrm{O}_{2_\textrm{peak}}$ for example through high intensity interval training (Laursen 2010) or on the improvement of Cc, e.g., through heavy strength training (Vikmoen et al. 2017). In this way, training time can be used more efficiently, which is especially relevant for young athletes due to their restricted schedules. In the future, longitudinal studies will be needed to identify the development of endurance performance following specific training based on an improvement in the physiological model predictors.

## Conclusion

Power output at LT2 can be accurately modeled using ${\dot{V}}\textrm{O}_{2_\textrm{peak}}$, %${\dot{V}}\textrm{O}_{2_\textrm{peak}}$ at LT2, and Cc for young cycling athletes of both sexes, with determination of Cc at 90% of LT2 providing the highest agreement. Of the physiological model predictors, ${\dot{V}}\textrm{O}_{2_\textrm{peak}}$ showed the largest influence on power output at LT2 in both sexes, ${\textrm{Cc}}_{\%{\rm LT}2}$ had a large influence only in female cyclists, and %${\dot{V}}\textrm{O}_{2_\textrm{peak}}$ at LT2 made only a minor contribution, while ${\dot{c}}La_\textrm{max}$ did not contribute significantly at all.

## Data Availability

The datasets generated and analyzed during the current study are available from the corresponding author on reasonable request.

## Abbreviations

bLa: Blood lactate concentration [mmol⋅L^–1^]
C: Exercise economy
Cc: Oxygen cost of cycling [mL⋅min^–1^⋅W^–1^]
${\textrm{Cc}}_{\textrm{fix}}$: Oxygen cost of cycling determined at a fixed power of 3 W⋅kg^–1^ [mL⋅min^–1^⋅W^–1^]
${\textrm{Cc}}_{\%{\dot{V}}\textrm{O}_{2_\textrm{peak}}}$: Oxygen cost of cycling determined at 75% of maximal oxygen uptake [mL⋅min^–1^⋅W^–1^]
${\textrm{Cc}}_{\%{\rm LT}2}$: Oxygen cost of cycling determined at 90% of lactate threshold 2 [mL⋅min^–1^⋅W^–1^]
${\dot{c}}La_\textrm{max}$: Maximal lactate accumulation rate [mmol⋅L^–1^⋅s^–1^]
GE: Gross efficiency [%]
GE_fix_: Gross efficiency determined at a fixed power output of 3 W. kg^−1^ [%]
GE_%_${\dot{V}}\textrm{O}_{2}$_peak_: Gross efficiency determined at 75% of maximal oxygen uptake [%]
GE_%LT2_: Gross efficiency determined at 90% of lactate threshold 2 [%]
ICC: Intra-class correlation coefficient
LT1: Lactate threshold 1
LT2: Lactate threshold 2
MLSS: Maximal lactate steady state
_mod_LT2: Modeled power corresponding to lactate threshold 2 [W]
_mod_LT2_fix_: Power corresponding to lactate threshold 2 modeled using oxygen cost of cycling determined at a fixed power of 3 W⋅kg^–1^ [W]
$_{\textrm{mod}}{\textrm{LT2}}_{\% {\dot{V}}\textrm{O}_{2_\textrm{peak}}}$: Power corresponding to lactate threshold 2 modeled using oxygen cost of cycling determined at 75% of maximal oxygen uptake [W]
_mod_LT2_%LT2_: Power corresponding to lactate threshold 2 modeled using oxygen cost of cycling determined at 90% of determined lactate threshold 2 [W]
${\dot{V}}\textrm{O}_{2}$: Oxygen uptake [mL⋅min^–1^]
${\dot{V}}\textrm{O}_{2_\textrm{peak}}$: Maximal oxygen uptake [mL⋅min^–1^]
%${\dot{V}}\textrm{O}_{2_\textrm{peak}}$: Fractional utilization of maximal oxygen uptake [%]

## References

[CR1] Besson T, Macchi R, Rossi J, Morio CYM, Kunimasa Y, Nicol C, Millet GY (2022) Sex differences in endurance running. Sports Med 52(6):1235–1257. 10.1007/s40279-022-01651-w 35122632 10.1007/s40279-022-01651-w

[CR2] Boisseau N, Delamarche P (2000) Metabolic and hormonal responses to exercise in children and adolescents. Sports Med 30(6):405–422. 10.2165/00007256-200030060-00003 11132123 10.2165/00007256-200030060-00003

[CR3] Borgen NT (2018) Running performance, VO2max, and running economy: the widespread issue of endogenous selection bias. Sports Med 48(5):1049–1058. 10.1007/s40279-017-0789-9 28983866 10.1007/s40279-017-0789-9

[CR4] Caen K, Poole DC, Vanhatalo A, Jones AM (2024) Critical power and maximal lactate steady state in cycling: ’’watts’’ the difference? Sports Med 54(10):2497–2513. 10.1007/s40279-024-02075-4 39196486 10.1007/s40279-024-02075-4

[CR5] Capelli C (1999) Physiological determinants of best performances in human locomotion. Eur J Appl Physiol 80(4):298–307. 10.1007/s004210050596 10.1007/s004210050596 10483799

[CR6] Clark CCT, Draper SB (2019) A detailed comparison of oxygen uptake kinetics at a range of exercise intensities. Motriz Revista de Educação Física. 25(1):. 10.1590/S1980-6574201900010010

[CR7] Costill DL, Branam G, Eddy D, Sparks K (1971) Determinants of marathon running success. Internationale Zeitschrift Für Angewandte Physiologie Einschließlich Arbeitsphysiologie 29(3):249–254. 10.1007/BF01100536 5565592 10.1007/BF01100536

[CR8] De Pauw K, Roelands B, Cheung SS, de Geus B, Rietjens G, Meeusen R (2013) Guidelines to classify subject groups in sport-science research. Int J Sports Physiol Perform 8(2):111–122. 10.1123/ijspp.8.2.111 23428482 10.1123/ijspp.8.2.111

[CR9] Diaz-Canestro C, Montero D (2019) Sex dimorphism of VO_2max_ trainability: a systematic review and meta-analysis. Sports Med 49(12):1949–1956. 10.1007/s40279-019-01180-z 31494865 10.1007/s40279-019-01180-z

[CR10] Faude O, Kindermann W, Meyer T (2009) Lactate threshold concepts: how valid are they? Sports Med 39(6):469–490. 10.2165/00007256-200939060-00003 19453206 10.2165/00007256-200939060-00003

[CR11] Haase R, Dunst AK, Nitzsche N (2024) The influence of pedaling frequency on blood lactate accumulation in cycling sprints. Int J Sports Med 45(8):608–615. 10.1055/a-2255-5254 38648800 10.1055/a-2255-5254

[CR12] Heck H, Schulz H, Bartmus U (2003) Diagnostics of anaerobic power and capacity. Eur J Sport Sci 3(3):1–23. 10.1080/17461390300073302

[CR14] Hopker J, Passfield L, Coleman D, Jobson S, Edwards L, Carter H (2009) The effects of training on gross efficiency in cycling: a review. Int J Sports Med 30(12):845–850. 10.1055/s-0029481-1237712 19941249 10.1055/s-0029-1237712

[CR13] Hopker J, Jobson S, Carter H, Passfield L (2010) Cycling efficiency in trained male and female competitive cyclists. J Sports Sci Med 9(2):332–337 24149704 PMC3761728

[CR15] Hunter GR, Bamman MM, Larson-Meyer DE, Joanisse DR, McCarthy JP, Blaudeau TE, Newcomer BR (2005) Inverse relationship between exercise economy and oxidative capacity in muscle. Eur J Appl Physiol 94(5–6):558–568. 10.1007/s00421-005-1370-z 15959800 10.1007/s00421-005-1370-z

[CR16] Iannetta D, Ingram CP, Keir DA, Murias JM (2021) Methodological reconciliation of CP and MLSS and their agreement with the maximal metabolic steady state. Med Sci Sports Exerc 54(4):622–632. 10.1249/mss.0000000000002831 10.1249/MSS.0000000000002831 34816811

[CR17] Ingham S, Whyte G, Jones K, Nevill A (2002) Determinants of 2,000 m rowing ergometer performance in elite rowers. Eur J Appl Physiol 88(3):243–246. 10.1007/s00421-002-0699-9 12458367 10.1007/s00421-002-0699-9

[CR18] Ingjer F (1991) Maximal oxygen uptake as a predictor of performance ability in women and men elite cross-country skiers. Scand J Med Sci Sports 1(1):25–30. 10.1111/j.1600-0838.1991.tb00267.x

[CR19] Jamnick NA, Botella J, Pyne DB, Bishop DJ (2018) Manipulating graded exercise test variables affects the validity of the lactate threshold and VO_2peak_. PLoS One 13(7):e0199794. 10.1371/journal.pone.0199794 30059543 10.1371/journal.pone.0199794 PMC6066218

[CR20] Jeukendrup AE, Wallis GA (2005) Measurement of substrate oxidation during exercise by means of gas exchange measurements. Int J Sports Med 26(Suppl 1):S28–S37. 10.1055/s-2004-830512 15702454 10.1055/s-2004-830512

[CR21] Ji S, Keller S, Zwingmann L, Wahl P (2023) Modeling lactate threshold in young squad athletes: influence of sex, maximal oxygen uptake, and cost of running. Eur J Appl Physiol 123(3):573–583. 10.1007/505s00421-022-05084-1 36411398 10.1007/s00421-022-05084-1 PMC9941268

[CR22] Johansen J-M, Sunde A, Helgerud J, Støren Ø (2022) Relationships between maximal aerobic speed, lactate threshold, and double poling velocity at lactate threshold in cross-country skiers. Front Physiol 13:829758. 10.3389/fphys.2022.829758 35295565 10.3389/fphys.2022.829758 PMC8918826

[CR23] Joyner MJ (1991) Modeling: optimal marathon performance on the basis of physiological factors. J Appl Physiol 70(2):683–687. 10.1152/jappl.1991.70.2.683 2022559 10.1152/jappl.1991.70.2.683

[CR24] Joyner MJ, Coyle EF (2008) Endurance exercise performance: the physiology of champions. J Physiol 586(1):35–44. 10.1113/jphysiol.2007.143834 17901124 10.1113/jphysiol.2007.143834 PMC2375555

[CR25] Koo TK, Li MY (2016) A guideline of selecting and reporting intraclass correlation coefficients for reliability research. J Chiropr Med 15(2):155–163. 10.1016/j.jcm.2016.02.012 27330520 10.1016/j.jcm.2016.02.012 PMC4913118

[CR26] Lacour J-R, Bourdin M (2015) Factors affecting the energy cost of level running at submaximal speed. Eur J Appl Physiol 115(4):651–673. 10.1007/s00421-015-3115-y 25681108 10.1007/s00421-015-3115-y

[CR27] Laursen PB (2010) Training for intense exercise performance: high-intensity or high-volume training? Scand J Med Sci Sports 20(Suppl 2):1-10. 10.1111/j.1600-0838.2010.01184.x 20840557 10.1111/j.1600-0838.2010.01184.x

[CR28] Lucía A, Hoyos J, Perez M, Santalla A, Chicharro JL (2002) Inverse relationship between VO2max and economy/efficiency in world-class cyclists. Med Sci Sports Exerc 34(12):2079–2084. 10.1249/01.MSS.0000039306.92778.DF 12471319 10.1249/01.MSS.0000039306.92778.DF

[CR29] Lundby C, Montero D, Gehrig S, Andersson Hall U, Kaiser P, Boushel R, Madsen K (2017) Physiological, biochemical, anthropometric, and biomechanical influences on exercise economy in humans. Scand J Med Sci Sports 27(12):1627–1637. 10.1111/sms.12849 28164383 10.1111/sms.12849

[CR30] Marín-Pagán C, Dufour S, Freitas TT, Alcaraz PE (2021) Performance profile among age categories in young cyclists. Biology 10(11):1196. 10.3390/biology10111196 34827189 10.3390/biology10111196 PMC8614687

[CR31] Mattioni Maturana F, Keir DA, McLay KM, Murias JM (2016) Can measures of critical power precisely estimate the maximal metabolic steady-state? Appl Physiol Nutr Metab 41(11):1197–1203. 10.1139/apnm-2016-0248 27819154 10.1139/apnm-2016-0248

[CR32] McLaughlin JE, Howley ET, Bassett DR, Thompson DL, Fitzhugh EC (2010) Test of the classic model for predicting endurance running performance. Med Sci Sports Exerc 42(5):991–997. 10.1249/mss.0b013e3181c0669d 19997010 10.1249/MSS.0b013e3181c0669d

[CR33] Meyler S, Bottoms L, Wellsted D, Muniz-Pumares D (2023) Variability in exercise tolerance and physiological responses to exercise prescribed relative to physiological thresholds and to maximum oxygen uptake. Exp Physiol 108(4):581–594. 10.1113/EP090878 36710454 10.1113/EP090878 PMC10103872

[CR34] Midgley AW, McNaughton LR, Polman R, Marchant D (2007) Criteria for determination of maximal oxygen uptake. Sports Med 37(12):1019–1028. 10.2165/00007256-200737120-00002 18027991 10.2165/00007256-200737120-00002

[CR35] Mukaka M (2012) Statistics corner: a guide to appropriate use of correlation in medical research. Malawi Med J 24(3):69–71 23638278 PMC3576830

[CR36] Niemeyer M, Gündisch M, Steinecke G, Knaier R, Beneke R (2022) Is the maximal lactate steady state concept really relevant to predict endurance performance? Eur J Appl Physiol 122(10):2259–2269. 10.1007/s00421-022-05001-6 35849182 10.1007/s00421-022-05001-6

[CR38] Quittmann OJ, Schwarz YM, Mester J, Foitschik T, Abel T, Strüder HK (2020) Maximal lactate accumulation rate in all-out exercise differs between cycling and running. Int J Sports Med 42(4):314–322. 10.1055/a-1273-7589 33137832 10.1055/a-1273-7589

[CR37] Quittmann OJ, Foitschik T, Vafa R, Freitag FJ, Sparmann N, Nolte S, Abel T (2023) Is maximal lactate accumulation rate promising for improving 5000-m prediction in running? Int J Sports Med 44(4):268–279. 10.1055/a-1958-3876 36529130 10.1055/a-1958-3876 PMC10072929

[CR39] Ray-Mukherjee J, Nimon K, Mukherjee S, Morris DW, Slotow R, Hamer M (2014) Using commonality analysis in multiple regressions: a tool to decompose regression effects in the face of multicollinearity. Methods Ecol Evol 5(4):320–328. 10.1111/2041-210X.12166

[CR40] Sabater-Pastor F, Faricier R, Metra M, Murias JM, Brownstein CG, Millet GY (2022) Changes in cost of locomotion are higher after endurance cycling than running when matched for intensity and duration. Med Sci Sports Exerc 55(3):389–397. 10.1249/mss.0000000000003059 36251372 10.1249/MSS.0000000000003059

[CR41] Scharhag-Rosenberger F, Meyer T, Gäßler N, Faude O, Kindermann W (2010) Exercise at given percentages of VO2max: heterogeneous metabolic responses between individuals. J Sci Med Sport 13(1):74–79. 10.1016/j.jsams.2008.12.626 19230766 10.1016/j.jsams.2008.12.626

[CR42] Schünemann F, Park S-Y, Wawer C, Theis C, Yang W-H, Gehlert S (2023) Diagnostics of la.max and glycolytic energy contribution indicate individual characteristics of anaerobic glycolytic energy metabolism contributing to rowing performance. Metabolites 13(3):317. 10.3390/metabo13030317 36984757 10.3390/metabo13030317 PMC10056884

[CR43] Sidossis L, Horowitz J, Coyle E (1992) Load and velocity of contraction influence gross and delta mechanical efficiency. Int J Sports Med 13(5):407–411. 10.1055/s-2007-1021289 1521959 10.1055/s-2007-1021289

[CR45] Støa EM, Støren Ø, Enoksen E, Ingjer F (2010) Percent utilization of VO_2max_ at 5-km competition velocity does not determine time performance at 5 km among elite distance runners. J Strength Condit Res 24(5):1340–1345. 10.1519/JSC.0b013e3181cc5f7b 10.1519/JSC.0b013e3181cc5f7b 20386483

[CR44] Støa EM, Helgerud J, Rønnestad BR, Hansen J, Ellefsen S, Støren Ø (2020) Factors influencing running velocity at lactate threshold in male and female runners at different levels of performance. Front Physiol 11:585267. 10.3389/fphys.2020.585267 33250778 10.3389/fphys.2020.585267 PMC7672120

[CR47] Støren Ø, Ulevåg K, Larsen MH, Støa EM, Helgerud J (2013) Physiological determinants of the cycling time trial. J Strength Condit Res 27(9):2366–2373. 10.1519/JSC.0b013e31827f5427 10.1519/JSC.0b013e31827f5427 23238091

[CR46] Støren Ø, Rønnestad BR, Sunde A, Hansen J, Ellefsen S, Helgerud J (2014) A time-saving method to assess power output at lactate threshold in well-trained and elite cyclists. J Strength Condit Res 28(3):622–629. 10.1519/JSC.0b013e3182a73e70 10.1519/JSC.0b013e3182a73e70 23942166

[CR48] Vikmoen O, Rønnestad BR, Ellefsen S, Raastad T (2017) Heavy strength training improves running and cycling performance following prolonged submaximal work in well-trained female athletes. Physiol Rep 5(5):e13149. 10.14814/phy2.13149 28292885 10.14814/phy2.13149 PMC5350167

[CR49] Wackerhage H, Gehlert S, Schulz H, Weber S, Ring-Dimitriou S, Heine O (2022) Lactate thresholds and the simulation of human energy metabolism: contributions by the cologne sports medicine group in the 1970s and 1980s. Front Physiol 13:899670. 10.3389/fphys.2022.899670 35936918 10.3389/fphys.2022.899670 PMC9353623

[CR50] Yang W-H, Meixner BJ, Sperlich B (2024) Uncertainty in determining the optimal test duration for maximal rate of lactate accumulation during all-out sprint cycle ergometry. Eur J Appl Physiol 10(124):3147–3148. 10.1007/s00421-024-05506-2 10.1007/s00421-024-05506-2 PMC11467032 38809479

[CR51] Zwingmann L, Strütt S, Martin A, Volmary P, Bloch W, Wahl P (2019) Modifications of the dmax method in comparison to the maximal lactate steady state in young male athletes. Phys Sportsmed 47(2):174–181. 10.1080/00913847.2018.1546103 30408426 10.1080/00913847.2018.1546103

