# Correction: Modeling lactate threshold in cycling—influence of sex, maximal oxygen uptake, and cost of cycling in young athletes

**DOI:** 10.1007/s00421-025-05890-3

**Published:** 2025-08-04

**Authors:** Jonas Fischer, Finn Hävecker, Sanghyeon Ji, Patrick Wahl, Sebastian Keller

**Affiliations:** 1 Department of Exercise Physiology, German Sport University, Cologne, Germany; 2 German Research Centre of Elite Sport, German Sport University, Cologne, Germany

**Correction: European Journal of Applied Physiology** 10.1007/s00421-025-05744-y

In the original version of this article, duplicated "+" should be replaced by a space in the first and seventh row of table 2 under the column "Proportional bias regression line (*p*-value)’’.

In Fig. 4 caption, subscript VO should be online in the equation $\dot{V}$O_2peak_ and figure 4 caption should have read as

Fig. 4 Scatter plots showing the Spearman’s rank correlation ρ between age and (**a**) maximal oxygen uptake ($\dot{V}$O_2peak_), (**b**) Oxygen cost of cycling determined at 90% of power output at lactate threshold 2 ( Cc%LT2 ), (**c**) fractional utilization of $\dot{V}$O_2peak_ at lactate threshold 2, (**d**) power output at lactate threshold 2 (LT2) determined using the modified maximal deviation method, (**e**) modeled power output at LT2 using oxygen cost of cycling at 90% of power output at LT2, and (**f**) the difference between modeled and experimentally determined power output at LT2. Individual data of male (*N* = 61) and female (*N* = 22) athletes are presented by blue triangles and red circles, respectively. The straight black line represents the linear regression

The original article has been corrected.

